# Impact of Neurorehabilitation on Functional Recovery in patients with Intradural Spinal Cord Tumors: A retrospective analysis in a resource-limited setting

**DOI:** 10.12669/pjms.41.13(PINS-NNOS).13429

**Published:** 2025-12

**Authors:** Zahra Khalid, Hafza Bushra Razaq, Humna Tariq, Amina, Samra Saleem

**Affiliations:** 1 Zahra Khalid Ch, BSPT, T-DPT, MS/MPhil. Chief Physiotherapist, Punjab Institute of Neurosciences, Lahore, Pakistan; 2 Hafza Bushra Razaq, DPT, MSPT- OMPT. Physiotherapist, Punjab Institute of Neurosciences, Lahore, Pakistan; 3 Humna Tariq, DPT. Physiotherapist, Punjab Institute of Neurosciences, Lahore, Pakistan; 4 Amina, DPT, MSPT- NMPT. Physiotherapist, Punjab Institute of Neurosciences, Lahore, Pakistan; 5 Samra Saleem, DPT, MSPT- WHPT. Physiotherapist, Punjab Institute of Neurosciences, Lahore, Pakistan

**Keywords:** Neurorehabilitation, Spinal Cord Neoplasms, Intra-dural space occupying lesions, Muscle strength, Functional recovery, Activities of daily living

## Abstract

**Objective:**

To assess the functional outcomes in patients with intradural spinal neoplasms following neurorehabilitation.

**Methodology:**

This retrospective case series was conducted in the Physiotherapy Department of Punjab Institute of Neurosciences, from July 2024 to December 2024. Data was collected from medical records of patients who underwent surgical resection of intradural spinal cord space-occupying lesions (SOL) followed by neurorehabilitation. Neurorehabilitation in this study comprised individualized, structured programs incorporating evidence-based manual therapy techniques, neurodevelopmental approaches, and task-specific training, delivered by specialized physical therapists. The treatment plans were designed according to patient needs and focused on restoring mobility, reducing spasticity, improving muscle strength, and enhancing independence in daily activities. Functional outcomes were assessed using the Numeric Pain Rating Scale (NPRS), Modified Ashworth Scale (MAS), Oxford Manual Muscle Testing (MMT), and Spinal Cord Independence Measure (SCIM). Assessments were performed following neurorehabilitation.

**Results:**

A total of 10 patients were included, with a mean age of 38.33 ± 23.71 years; 80% (8) were female. Most participants, 70% (7) had intradural extramedullary (IDEM) tumors, while 30% (3) had intradural intramedullary (IDIM) tumors. Post-rehabilitation, NPRS scores significantly decreased from 6.70 ± 0.95 to 2.50 ± 0.85. MAS scores improved from 2.80 ± 0.42 to 1.60 ± 0.52, indicating reduced spasticity. MMT scores increased from 2.00 ± 0.47 to 3.30 ± 0.48, demonstrating improved muscle strength. SCIM scores improved from 31.40 ± 4.99 to 63.50 ± 8.89, highlighting enhanced functional independence.

**Conclusion:**

Neurorehabilitation particularly early initiation and frequent, structured sessions using manual therapy and neurodevelopmental techniques significantly improves pain levels, muscle tone, strength, and functional independence in patients following surgical resection of intradural spinal tumors. Early and structured rehabilitation programs do play a crucial role in optimizing post-operative recovery in these patients.

## INTRODUCTION

Neuro-oncology refers to the diagnosis and management of primary and secondary nervous system tumors.^1^ Individuals with spinal cord tumors (i.e. benign or malignant) experience significant neurological deficits.^2^ Spinal intradural tumors are of two categories: intramedullary and extramedullary. The most common location 71.1% was the thoracic region followed by the cervical and lumbar regions.^3^ Diagnostic investigations such as Magnetic Resonance Imaging with contrast and definitive histopathology are used to make a provisional diagnosis.^4^ Although surgery for spinal tumors generally results in favorable outcomes, it may still be associated with certain postoperative deficits.^5^ These tumors can lead to a range of impairments, including motor, sensory, and autonomic dysfunctions, which necessitate targeted rehabilitation interventions.^6^

Major deficiencies include progressive motor deficits, cancer-associated cachexia (CAC), sensory deficits, spasticity, pain, fatigue, pressure ulcers, bladder and bowel loss, deep vein thrombosis which need neurorehabilitation.^7^ The process of helping someone who has been disabled by a tumor (or treatments) to improve their symptoms and maximize their functional independence is known as neuro-oncological rehabilitation.^8^ Neuro-rehabilitation strategies used such as modalities for neuropathic pain like Transcutaneous electrical nerve stimulation (TENS), stretches for spasticity, strengthening or ROM exercises for progressive muscular weakness and to access functional independence in inpatient and outpatient.^9^

Most used scale for spasticity is Modified Ashworth Scale (MAS).^10^ Pain numeric rating scale for patient pain response.^11^ Oxford Manual Muscle Test (MMT) is a standardized method for evaluating muscle strength on a 0–5 scale, which can indirectly reflect muscle weakness when reduced scores are observed is used for muscle weakness.^12^ Functional independence is evaluated by using Spinal Cord Injury Measure (SCIM) tool.^13^ Although previous studies have highlighted the role of rehabilitation in spinal injury recovery, limited data exist regarding the impact of neurorehabilitation following surgical resection of intradural spinal cord tumors, particularly in resource-limited settings. This study aims to address this gap by evaluating the effect of structured neurorehabilitation on post-operative complications in patients with intradural spinal cord tumors. The objective pf this study was to contribute evidence for integrating early and targeted rehabilitation into routine care for spinal tumor patients, especially in developing healthcare systems.

## MEHODOLOGY

This is a retrospective case series study. It was conducted in the Physiotherpy Department of Punjab Institute of Neurosciences. Study duration was six months from July 2024 to December 2024. Data was collected through non-probability convenience sampling for all consecutive cases of intradural spinal cord SOL who were subjected to surgical removal of the SOL. Sample size of the study was 10.

All patients underwent individualized multidisciplinary neurorehabilitation program including physical therapy, occupational therapy, and medical treatment specific to neurorehabilitation. Exercise programs began within first postoperative week, consisting of evidence-based manual therapy techniques, stretching, strengthening, range-of-motion exercises, and neurodevelopmental training. Sessions were delivered by specialized physical therapists and conducted two to three times per day as needed. No advanced neuromodulation devices were available; instead, available resources included mats, exercise bands and parallel bars. Physical agents such as cryotherapy and thermotherapy as well as electrotherapeutic modalities, were not incorporated.

### Ethical Approval:

It was given by the institutional review board (IRB) of Punjab Institute of Neurosciences via reference no. 2078/IRB/PINS/Approval/2025, dated: February 20, 2025).

### Inclusion criteria

- All patients who had intradural spinal cord SOL and went through surgical resection of SOL along with neurorehabilitation were included in the study.
- Patients of all ages and genders.

### Exclusion criteria

- Patients who have systemic pathological conditions (spinal TB, complete cord compression).
- Patients with history of recent trauma.
- Patients having intraoperative or postoperative complications leading to death.
- Patients with incomplete operative notes and follow-up notes were excluded.

Data was collected from patients’ medical records. This included patient files, clinical notes, post-operative stay in the hospital, post-operative clinic follow-up in OPD. Patients were followed for up to six weeks post-discharge to assess short-term outcomes; no long-term follow-up data was available. Potential confounding factors, such as nutritional status and advanced age, were considered when interpreting results. Depending on the availability of data, outcomes were assessed pre and post neurorehabilitation by using the Numeric Pain Rating Scale (NPRS), Modified Ashworth Scale (MAS), Oxford Manual Muscle Testing (MMT), and Spinal Cord Independence Measure (SCIM).

### Data analysis technique:

Google Forms (Google Inc., USA) were used for data collection and analysis. Categorical data was presented as frequencies and percentages. Quantitative data was presented as means with SDs (standard deviation). Only descriptive statistics were used in this study.

## RESULTS

The age distribution of the 10 patients had a mean age of 38.33 ± 23.71 years, indicating a diverse age group with considerable variability among the participants. Out of the 10 patients, 20% (2) were male, and 80% (8) were female, indicating a higher representation of female participants in the study population. All cases included in the study were intradural spinal tumors. Among these, IDIM were found in 30% (3) of the patients, while IIDEM were observed in 70% (7) of the patients. This distribution highlights the predominance of IDEM cases within the study group. The site of lesion distribution showed that 40% (4) of the lesions were in the cervical region, 30% (3) were in the thoracic region, and 30% (3) were in the lumbar region. This distribution indicates that cervical lesions were the most common in the study population.

The Numeric Pain Rating Scale (NPRS) scores show a significant reduction in pain intensity following rehabilitation. The mean pre-rehabilitation score was 6.70 ± 0.95, which decreased to 2.50 ± 0.85 post-rehabilitation. Patients 3 and 10 showed particularly marked pain reduction, from 7 to 2 and from 8 to 3 respectively, suggesting strong analgesic effects of the rehabilitation interventions in high-pain cases. (Table-I) The Modified Ashworth Scale (MAS) scores, which assess spasticity, demonstrated a notable improvement after rehabilitation. The mean pre-rehabilitation score was 2.80 ± 0.42, reducing to 1.60 ± 0.52 post-rehabilitation. Patients 3, 7, and 8 improved most significantly, each reducing from a score of 3 to 1, indicating successful spasticity management through the applied techniques. (Table-II)

**Table-I:** Pre-rehabilitation and post-rehabilitation Numeric Pain Rating Scale (NPRS) scores along with the mean and standard deviation.

Patients	Pre-Rehab NPRS Score (n)	Post-Rehab NPRS Score (n)
Patient 1	6	2
Patient 2	5	1
Patient 3	7	2
Patient 4	8	4
Patient 5	7	3
Patient 6	7	3
Patient 7	6	2
Patient 8	6	2
Patient 9	7	3
Patient 10	8	3
Mean ± SD (N)	6.70 ± 0.95	2.50 ± 0.85

Legend: N = Total number of patients, n = Individual score, SD = standard deviation.

**Table-II:** Pre-rehabilitation and post-rehabilitation Modified Ashworth Scale (MAS) scores along with the mean and standard deviation

Patients	Pre-Rehab MAS Scores (n)	Post-Rehab MAS Scores (n)
Patient 1	2	1
Patient 2	2	2
Patient 3	3	1
Patient 4	3	2
Patient 5	3	2
Patient 6	3	2
Patient 7	3	1
Patient 8	3	1
Patient 9	3	2
Patient 10	3	2
Mean ± SD (N)	2.80 ± 0.42	1.60 ± 0.52

Legend: N = Total number of patients, n = Individual score, SD = standard deviation.

The Oxford Manual Muscle Testing (MMT) scores reflect an improvement in muscle strength post-rehabilitation. The mean pre-rehabilitation score was 2.00 ± 0.47, which increased to 3.30 ± 0.48 post-rehabilitation. Notably, Patient 3 improved from grade Two to Four., Patient four from one to three, and Patient seven from two to four, indicating substantial recovery even in cases with low initial strength levels. This improvement indicates enhanced muscle strength, highlighting the positive impact of the rehabilitation program on muscle functionality. (Table-III) The Spinal Cord Independence Measure (SCIM) scores, which evaluate functional independence, showed a marked improvement post-rehabilitation. The mean pre-rehabilitation score was 31.40 ± 4.99, which significantly increased to 63.50 ± 8.89. The greatest individual improvements were seen in patients seven and eight each with a 40-point increase, confirming substantial gains in daily living independence. (Table-IV)

**Table III:** Pre-rehabilitation and post-rehabilitation Oxford Manual Muscle Testing (MMT) scores along with the mean and standard deviation.

Patients	Pre-Rehab MMT Scores (n)	Post-Rehab MMT Scores (n)
Patient 1	3	4
Patient 2	2	3
Patient 3	2	4
Patient 4	1	3
Patient 5	2	3
Patient 6	2	3
Patient 7	2	4
Patient 8	2	3
Patient 9	2	3
Patient 10	2	3
Mean ± SD (N)	2.00 ± 0.47	3.30 ± 0.48

Legend: N = Total number of patients, n = Individual score, SD = standard deviation.

**Table-IV:** Pre-rehabilitation and post-rehabilitation Spinal Cord Independence Measure (SCIM) scores along with the mean and standard deviation.

Patients	Pre-Rehab SCIM Scores (n)	Post-Rehab SCIM Scores (n)
Patient 1	39	72
Patient 2	26	49
Patient 3	33	71
Patient 4	24	56
Patient 5	33	59
Patient 6	24	54
Patient 7	34	74
Patient 8	33	73
Patient 9	34	62
Patient 10	34	65
Mean ± SD (N)	31.40 ± 4.99	63.50 ± 8.89

Legend: N = Total number of patients, n = Individual score, SD = standard deviation.

## DISCUSSION

In a setting, where resources are limited, specifically in terms of advanced rehabilitation equipment and neuromodulation devices, this study demonstrates the impact of neurorehabilitation in subjects with intra-dural spinal cord tumors. Pre-rehabilitation and post-rehabilitation scores showed significant reduction in pain and spasticity, gains in muscle strength and achievement of functional independence. These results are consistent with the existing body of literature that supports the role of a structured and goal-oriented rehabilitation program in improving quality of life of patients with spinal cord tumors.

Pain is a common complication in spinal cord tumor patients, often persists even after surgical interventions.^14^ There was a significant decrease in NPRS scores (i.e from 6.70 ± 0.95 pre-rehabilitation to 2.50 ± 0.85 post-rehabilitation). The pain reduced in severity from moderate to mild pain, suggesting the effectiveness of neurorehabilitation interventions. In particular, patients with higher baseline pain such as Patient 3 (7 to 2) and Patient 10 (8 to 3) demonstrated remarkable improvement, underscoring the program’s value for severe pain cases. Similar results have been reported by Patel et al., in 2025 showing considerable improvement in NPRS scores following a targeted rehabilitation program after excision of spinal tumor. The magnitude of improvement in our study was likely influenced by the fact that rehabilitation began within the first postoperative week and was delivered two to three times daily according to patient need, as compared to the study reported by Patel and colleagues, where rehabilitation program was started 1.5 weeks post-operatively.^15^ The difference in MAS scores (from 2.80 ± 0.42 pre-rehabilitation to 1.60 ± 0.52 post-rehabilitation) indicates that there was improvement in degree of spasticity in the subjects following neurorehabilitation interventions. It perfectly aligns with results of the study conducted by Saputtitada et al., in 2024, on the effect of stretching and other techniques of neuorehabilitation in reduction of muscle spasticity. Patients 3, 7, and 8 improved from MAS 3 to 1, representing substantial tone reduction. The study reported greater improvement than ours, likely because advanced techniques and equipment were available, whereas our setting relied exclusively on manual therapy techniques and basic rehabilitation tools.^16^

Muscle strength, as assessed using MMT scale, showed significant improvement i.e. from 2.00 ± 0.47 pre-rehabilitation to 3.30 ± 0.48 post-rehabilitation. As clarified earlier, the MMT is a standardized measure of muscle strength, not weakness, though reduced scores may indicate weakness. Individual gains such as Patient 3 (grade 2 to 4), Patient 4 (grade 1 to 3), and Patient 7 (grade 2 to 4) highlight the program’s ability to restore function even in severely impaired patients. Similarly, SCIM scores revealed considerable gains in functional independence i.e., from 31.40 ± 4.99 pre-rehabilitation to 63.50 ± 8.89 post-rehabilitation. Patients 7 and 8 demonstrated the largest individual functional recovery, each improving by 40 points. This aligns with Sakaguchi et al. (2024), who emphasized a multifaceted rehabilitation program to restore mobility and enhance quality of life.^17^ A retrospective study on spinal meningioma is in line with the results of this study and has shown comparable improvements in functional outcomes following a neuro-oncological rehabilitation program.^5^ However, in the current study, the difference in SCIM score outcomes was more statistically significant. This can be explained by the fact that rehabilitation intervention sessions were conducted more frequently (two to three times a day as per patient need) compared to the studies cited, where rehabilitation sessions were generally provided only once daily. This finding further implies that the success of a neurorehabilitation program also depends on the frequency and intensity of sessions.

While our discussion initially attributed some limitations in improvement to the absence of advanced equipment, it is important to acknowledge that evidence-based manual therapy techniques such as the Brunnstrom approach and the Carr & Shepherd concept are strongly supported by clinical research and can often match or exceed machine-based modalities in effectiveness. Our results affirm the value of these approaches, particularly in resource-limited settings.

The findings of the study have important clinical implications for neurorehabilitation in patients with spinal cord tumors. Given the significant improvement in pain management, muscle strength and functional independence,^18^ a multidisciplinary approach involving physical therapists, occupational therapists, and medical staff specialized in neurorehabilitation can optimize patient outcomes as reported by Rispoli et al.^19^ especially in settings where advanced neuromodulation devices are unavailable but basic equipment such as mats, parallel bars and exercise band is accessible.^20^

### Limitations and Future Directions:

Regardless of all these positive outcomes, this study had certain limitations. A key limitation is the exclusive use of manual techniques in rehabilitation program, without incorporating physical agents (cryotherapy, thermotherapy) or electro-therapeutic modalities.

Future research should explore individualized rehabilitation approaches keeping in mind different tumor categories and long-term functional outcomes to further refine rehabilitation strategies for these patients. Also, there should be an integration of advanced neuro-modulation devices and technologies, such as Transcutaneous nerve stimulator (TENS), Interferential Therapy, Resistance-based Vibration Therapy, Neuromuscular electronic stimulators (NMES etc.) and other equipment (e.g spirometers, tilt-tables, transfer boards etc.) that are widely being used worldwide. Additionally, there was a lack of long-term follow up data for the majority of patients. Although significant efforts were made to contact the subjects telephonically, a major portion was not reachable, making it challenging to assess the sustainability of the observed outcomes. Factors such as nutritional status and older age may have influenced recovery. The retrospective nature of the study, relatively small sample size and resource limitations may limit the generalizability of the results. Future studies should be done to see the effects of TENS, Transcranial magnetic stimulation (TMS) for neuroplasticity enhancement and Functional electronic stimulation (FES) for neuro-muscular re-education. Further researches should also investigate the relationship between pre-operative interventions and post-operative mobility in patients undergoing resection of intradural spinal cord tumor.

## CONCLUSION

In this short-term study, early and frequent manual therapy-based neurorehabilitation using available low-cost equipment was effective in improving pain, muscle tone, strength, and functional independence following intradural spinal tumor surgery. These findings are consistent with the existing literature, highlighting the importance of targeted rehabilitation in improving recovery outcomes.

### Authors Contributions:

**ZKC:** Concept and designed, critically reviewed and supervised the study.

**HBR:** Data analysis and data interpretation, critically reviewed the study.

**HT, Amina and SS:** Data acquisition and data interpretation, drafted the manuscript.

All the authors contributed to the final approval of the version to be published and in agreement to be accountable for all aspects of the work.
